# Clinical Outcomes of the Alternative GAMMA Regimen in Relapsed Germ Cell Tumours

**DOI:** 10.1002/cam4.71964

**Published:** 2026-05-19

**Authors:** Nasreen Abdul Aziz, Kenrick Ng, Prabhakar Rajan, Jonathan Shamash

**Affiliations:** ^1^ School of Medicine, School of Postgraduate Studies, Royal College of Surgeons in Ireland Dublin Ireland; ^2^ St Bartholomew's Hospital, Barts Health NHS Trust London UK; ^3^ Centre for Experimental Cancer Medicine, Barts Cancer Institute, Queen Mary University of London London UK; ^4^ Centre for Cancer Cell and Molecular Biology, Barts Cancer Institute, Queen Mary University of London London UK; ^5^ The Royal London Hospital, Barts Health NHS Trust London UK

## Abstract

**Background:**

Following promising results in a phase II trial evaluating GAMMA as salvage therapy for relapsed germ cell tumours (GCT) who had progressed on cisplatin‐based chemotherapy, the GAMMA regimen was adopted into clinical practice at St Bartholomew's Hospital. This study provides the largest real‐world evaluation of the GAMMA regimen to date, assessing its long‐term effectiveness in an unselected patient population.

**Methodology:**

This was a single‐centre retrospective study of patients who progressed following cisplatin‐based chemotherapy and were treated with GAMMA regimen between November 2012 and September 2023. Data collected included clinico‐pathological features, treatment details and prognostic risk scores which are International Germ Cell Cancer Collaborative Group (IGCCCG) risk classification and International Prognostic Factor Study Group (IPFSG) score. Progression‐free survival (PFS), overall survival (OS) and objective response rate (ORR) were assessed. Survival was estimated using the Kaplan–Meier method and compared using the log‐rank test.

**Results:**

A total of 69 patients were included with a median age of 39 years. Most were male (94%) and had an ECOG performance status of 0–1 (76%). Non‐seminomatous histology was observed in 80% and 74% had a gonadal primary tumour. BEP was the most common first‐line treatment (89%). Poor‐risk disease per IGCCCG criteria was seen in 41% while 39% were intermediate‐risk by IPFSG. 64% completed all planned chemotherapy cycles; 10% discontinued due to toxicity. Stem cell mobilisation was successful in 91%. The most frequent treatment response was partial response marker negative (PRm‐) (38%). The 2‐year PFS and OS rates were 31% and 49%, respectively. By IPFSG risk group, 2‐year PFS and OS ranged from 20%–50% and 20%–75%. Among patients with LDH ≥ 2.5xULN, 2‐year PFS was 38% and OS was 47%.

**Conclusions:**

GAMMA demonstrates acceptable tolerability and maintains meaningful survival outcomes, supporting its use as a dose‐intensified salvage treatment option for patients with relapsed GCT.

## 1 Background

Testicular germ cell tumours (GCT) are the most common malignancy in young adult men with a peak incidence between the ages of 15 and 35 [1, 2]. The majority of patients with testicular cancer are diagnosed at an early stage but approximately 11% present with metastatic disease at the time of initial diagnosis [3]. Although first‐line therapy is highly effective, approximately 20%–30% will experience disease relapse and subsequently require further lines of treatment [4, 5].

Several dose‐intense regimens have been developed in the united Kingdom (UK) for patients with poor‐risk germ cell tumours, including POMB/ACE (cisplatin, vincristine, methotrexate, bleomycin, actinomycin D, cyclophosphamide, etoposide) [6], CBOP/BEP (carboplatin, bleomycin, vincristine, cisplatin, doxorubicin, bleomycin, etoposide, cisplatin) [7] and GAMEC (G‐CSF, actinomycin D, methotrexate, etoposide, cisplatin) [8], all of which aim to improve outcomes through early chemotherapy intensification. At St Bartholomew's Hospital (SBH), we have previously shown that the GAMEC regimen achieved promising results in poor‐risk and relapsed patients but its use was limited by toxicity particularly in older individuals and those with high lactate dehydrogenase (LDH) [9]. The GAMMA regimen was developed as a modified version of GAMEC, replacing cisplatin with oxaliplatin and etoposide with paclitaxel with the goal of reducing toxicity while maintaining efficacy in patients with cisplatin‐resistant or poor‐prognosis disease.

The GAMMA regimen was investigated in a phase II study as a modified salvage treatment for patients with relapsed GCT who had progressed following cisplatin‐based chemotherapy [10]. This study cohort was predominantly intermediate‐poor risk patients, yet the regimen achieved an objective response rate (ORR) of 59%, with a 2‐year progression‐free survival (PFS) of 33% and overall survival (OS) not reached at the time of analysis. These results demonstrate that GAMMA can provide meaningful anti‐tumour activity and remains a potential curative option in this setting. Based on these findings, SBH has adopted the GAMMA regimen into clinical practice.

This study evaluates the real‐world outcomes of the GAMMA regimen in the treatment of relapsed GCT and represents the largest reported dataset to date examining its use. Importantly, it provides insights into the regimen's effectiveness in patients treated outside the context of a clinical trial. Additionally, the study assesses the feasibility of successful stem cell mobilisation following GAMMA regimen supporting its potential role as first salvage option without compromising subsequent high‐dose chemotherapy (HDCT). Finally, this analysis explores the prognostic impact of LDH, a recognised risk factor that has not previously been evaluated in the context of the GAMMA regimen.

## 2 Methodology

### 2.1 Methods and Patients

Single‐centre retrospective study was conducted in SBH including all patients with metastatic germ cell tumours who had received first‐line cisplatin‐etoposide chemotherapy and the GAMMA regimen between November 2012 and September 2023 with the data cutoff set at 31st July 2025. This cohort included patients from the original phase II study (*n* = 44), as well as all patients treated with the GAMMA regimen thereafter. After the trial closed to recruitment, additional patients received the GAMMA regimen as part of routine clinical care and provided standard written informed consent for treatment. Institutional approval was obtained from SBH (project number 14581).

### 2.2 Eligibility Criteria

Eligible participants were aged 16 to 65 years with histologically confirmed GCT of gonadal or extragonadal origin, who had previously received platinum‐based chemotherapy and experienced disease relapse or progression. Patients were excluded if they had prior exposure to oxaliplatin, methotrexate or actinomycin D and had an ECOG performance status of 4.

### 2.3 Data Collection

Data were gathered from SBH using electronic healthcare systems. The collected dataset included patient age, ECOG performance status, histology, primary tumour location, tumour markers (LDH, alpha‐fetoprotein (AFP) and beta‐human chorionic gonadotropin (b‐HCG)), number of chemotherapy cycles delivered, type of first‐line regimen, salvage chemotherapy administered after GAMMA, International Germ Cell Cancer Collaborative Group (IGCCCG) risk classification, International Prognostic Factor Study Group (IPFSG) score, reason for treatment discontinuation, response rate, incidence of relapse, survival status, and documented cause of death.

### 2.4 Treatment Regimen

Patients received chemotherapy administered intravenously in 21‐day cycles, for a total of three to four cycles. The regimen consisted of actinomycin D, methotrexate and paclitaxel on Day 1; oxaliplatin on Day 4; a combination of paclitaxel and oxaliplatin on Day 8 and a final dose of paclitaxel on Day 15. In addition, pegfilgrastim was administered subcutaneously on Day 3 of each cycle in accordance with the original GAMMA phase II protocol, to provide prophylactic support against chemotherapy‐induced myelosuppression and facilitate delivery of subsequent chemotherapy doses within the planned schedule.

**FIGURE 1:**
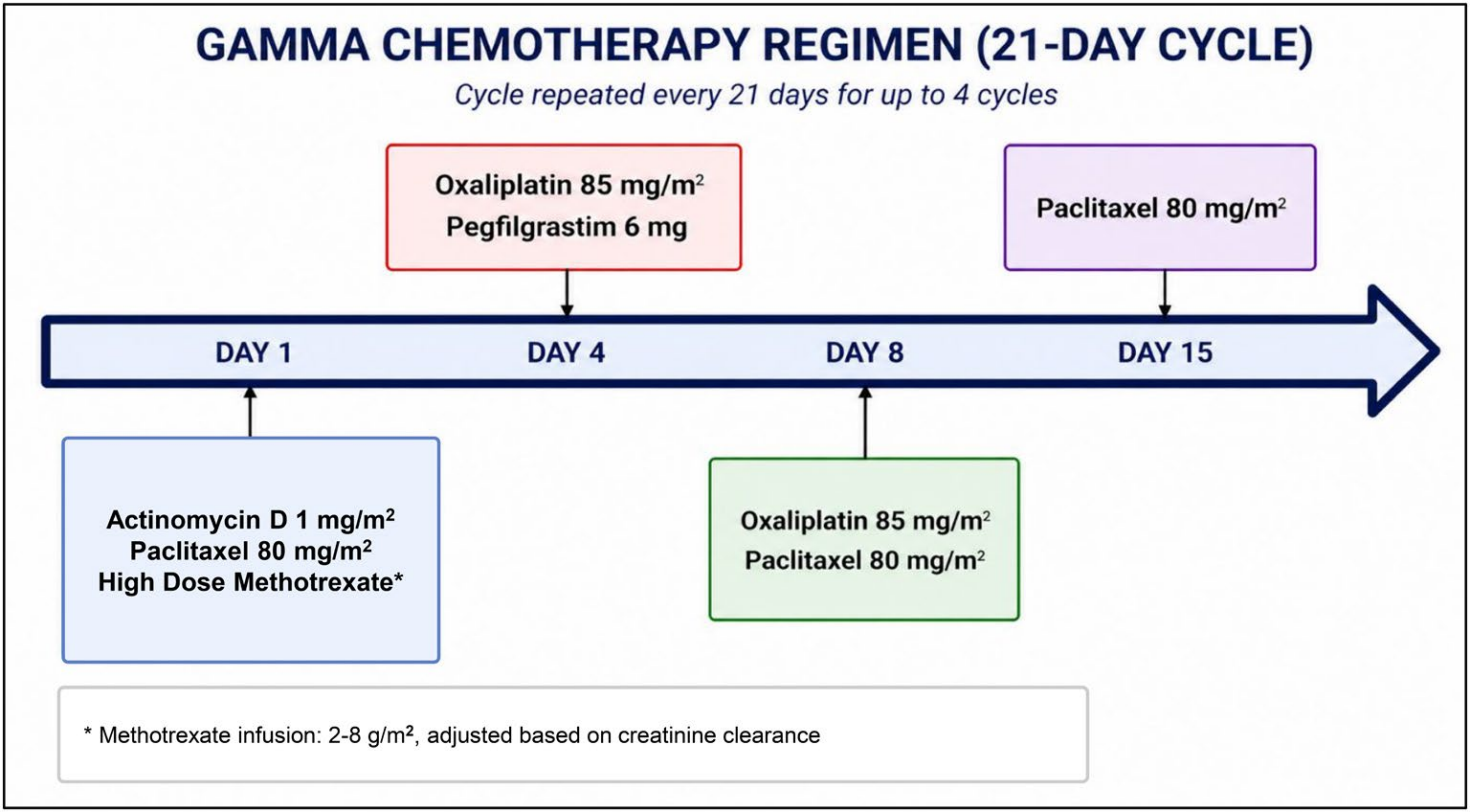
Schedule and dosing of the GAMMA chemotherapy regimen.

Subsequent treatment following GAMMA, including the use of HDCT, was not standardised and was based on institutional practice and individual patient factors, including prior treatment exposure, comorbidities, and clinician preference. The choice of HDCT regimen and number of cycles was determined on a case by case basis. The detailed drug schedule and dosing of the GAMMA regimen are summarised in Figure 1.

### 2.5 Follow Up

Patients were followed up every 2 months in the first year, with tumour markers monitored monthly if they had not normalised. In the second year, follow up was every 3 months and every 4 months in the third year. In Years 4 and 5, follow up occurred every 6 months and annually from Year 6 to 10. A CT scan was performed at the end of Year 1 and again at the end of Year 2. If residual radiological abnormalities were present post‐treatment, imaging was repeated every 4 to 6 months until stability was confirmed, with resection considered where appropriate. Follow up blood tests at each visit included full blood count, renal function, liver function tests and serum tumour markers (AFP, b‐HCG and LDH). The follow up schedule used in our cancer centre is based on the Anglian Germ Cell Cancer Collaborative Group (AGCCCG) supraregional testicular follow up schedule.

### 2.6 Statistical Analysis

Statistical analysis was conducted using R software (version 4.5.1). Kaplan–Meier survival analysis was used to estimate progression‐free survival (PFS) and overall survival (OS). PFS was defined as the time from the first day of treatment to either disease progression or death from any cause, whichever occurred first while OS was defined as the time from the first day of treatment until death from any cause. Survival curves were compared between IPFSG risk categories and baseline LDH level (< 2.5xULN vs. ≥ 2.5xULN) using the log‐rank test. Descriptive analysis was performed to examine the demographic characteristics.

Response to treatment was assessed based on radiological imaging and serum tumour marker evaluation following completion of treatment or at the time of treatment discontinuation. ORR was defined as the proportion of patients achieving a complete response (CR) or partial response with normalised tumour markers (PRm−). Marker‐positive partial responses (PRm+) were not included in the ORR calculation. Patients who were not evaluable for response, including those who discontinued treatment early due to toxicity, progression, or other reasons, were excluded from the ORR analysis.

## 3 Results

Data were collected from 69 patients with a median follow up of 71 months. The median age at diagnosis was 39 years (range 22–63). There were 65 (94%) male and 4 (6%) female patients. The majority had an ECOG performance status of 0–1 (*n* = 53, 76%), while 6 (8%) had ECOG 2–3. Histologically, 55 (80%) had non‐seminomatous germ cell tumours (NSGCT) followed by 13 (19%) with seminoma and 1 (1%) with teratoma.

The primary tumour site was gonadal in 55 patients (80%) and extragonadal in 13 (19%). Among the gonadal tumours, 52 (76%) originated in the testis and 3 (4%) in the ovary. Of the extragonadal tumours, 7 (10%) were in the mediastinum. A total of 61 patients (89%) received one prior lines of systemic therapy, 6 (8%) received two prior lines and 1 patient (1.5%) had received more than three prior lines. The most common first‐line chemotherapy regimen was bleomycin, etoposide and cisplatin (BEP). Other regimens included etoposide, ifosfamide and cisplatin (VIP), carboplatin AUC10 and GAMEC.

Based on the International Germ Cell Cancer Collaborative Group (IGCCCG) risk classification, 21 patients (30%) were classified as good risk, 12 (17%) as intermediate risk and 28 (41%) as poor risk. Upon relapse, patients were reclassified according to the International Prognostic Factor Study Group (IPFSG) score where 4 (6%) were categorised as very low risk, 12 (17%) as low risk, 27 (39%) as intermediate risk, 13 (19%) as high risk and 5 (7%) as very high risk. LDH levels at relapse were < 2.5xULN in 59 patients (86%), while 8 patients (12%) had LDH levels ≥ 2.5xULN. LDH data were missing for 2 of 69 patients (2%). Table 1 describe the study characteristics.

**TABLE 1:** Study characteristics of study cohort.

Baseline characteristics	*N* (%)
Gender	
Male	65 (94)
Female	4 (6)
Histology	
Seminoma	13 (19)
Non seminoma	55 (80)
Teratoma	1 (1)
ECOG	
0	30 (44)
1	22 (32)
2	3 (4)
3	3 (4)
NA	11 (16)
Primary side	
Gonadal^a^	55 (80)
Extragonadal^b^	13 (19)
Unknown	1 (1)
IGCCCG classifications	
Good	21 (30)
Intermediate	12 (17)
Poor	28 (41)
Unknown	8 (12)
Lines of treatment	
2	61 (89)
3	6 (8)
≥ 4	1 (1.5)
Unknown	1 (1.5)
LDH at relapse	
< 2.5xULN	59 (86)
≥ 2.5xULN	8 (12)
Unknown	2 (2)
IPFSG Score	
Very low	4 (6)
Low	12 (17)
Intermediate	27 (39)
High	13 (19)
Very high	5 (7)
NA	8 (12)

^a^ Testis 52 (76%), ovary 3 (4%).

^b^ Mediastinal 7 (10%).

The GAMMA delivery and outcome is described in Table 2. A total of 34 patients (49%) completed all four planned cycles of GAMMA chemotherapy. Fifteen patients (22%) received three cycles, 12 (17%) received two cycles and 7 (10%) received only one cycle.

**TABLE 2:** GAMMA Chemotherapy delivery and clinical outcomes.

GAMMA regimen	*N* (%)
Number of cycles	
1	7 (10)
2	12 (17)
3	15 (22)
4	34 (49)
NA	1 (2)
Reason for treatment discontinuation	
Complete treatment	44 (64)
Toxicity	7 (10)
Progressed	14 (20)
Others^a^	2 (3)
Unknown	2 (3)
Best treatment response	
CR	7 (10)
PRm−	26 (38)
PRm+	2 (3)
SD	5 (7)
PD	18 (26)
Not evaluable	8 (12)
Unknown	3 (4)
Post‐GAMMA relapse treatments	
HDCT	30 (68)
Palliative chemotherapy	4 (9)
CDCT	2 (5)
Others^b^	8 (18)

^a^ Declined treatment and were non‐compliant.

^b^ Declined chemotherapy (*n* = 1), not for treatment (*n* = 5), unknown (*n* = 2).

The most common reason for treatment discontinuation was completion of planned therapy (*n* = 44, 64%). Fourteen patients (20%) experienced disease progression during treatment, while 7 (10%) discontinued due to toxicity. Among those, three patients died from treatment‐related causes. Other reasons included treatment refusal or non‐compliance (*n* = 2, 3%).

In terms of best treatment response, the ORR is 48% with 7 patients (10%) achieved a CR, 26 (38%) had a PRm− and 2 (3%) had a PRm+. Stable disease (SD) was observed in 5 patients (7%) while 18 (26%) experienced disease progression (PD). Eight patients (12%) were not evaluable for response due to receiving only one cycle of treatment as a result of toxicity, non‐compliance, treatment refusal or treatment‐related death.

Following relapse (*n* = 44), 30 patients (68%) proceeded to HDCT with stem cell mobilisation successful in all cases. The most common regimens included high dose epirubicin/carboplatin/etoposide/melphalan (Epi‐CEM), topotecan/carboplatin/thiotepa (TopCat) and carboplatin/etoposide (CE). Four patients (9%) received palliative chemotherapy; three were unable to proceed to HDCT due to stem cell harvest failure and 1 had a successful harvest but progressed before receiving HDCT. Two patients (5%) received conventional‐dose chemotherapy (CDCT) both of whom were deemed unfit for stem cell harvest due to treatment‐related toxicity or mental health issues. Within the HDCT group, six patients (20%) remained alive and disease‐free following the GAMMA regimen. Stem cell mobilisation was successful in 31 out of 34 patients (91%) who underwent the procedure. Table 3 summarises the distribution of post‐GAMMA treatments and stem cell harvest feasibility.

**TABLE 3:** Distribution of post‐GAMMA treatments and feasibility of stem cell harvest.

Post‐GAMMA treatments	*n*	Key details	Stem cell harvests successful, *n* (%)	Alive and disease free, *n* (%)
HDCT	30	Epi‐CEM (*n* = 13), Top‐CAT (*n* = 8), CE (*n* = 9)	30 (100)	6 (20)
Palliative chemotherapy	4	3 stem cell harvests failed, 1 harvest successful but progressed before HDCT	1 (25)	0 (0)
CDCT	2	Not fit for stem cell harvest (toxicity/mental health)	0 (0)	0 (0)

At the time of analysis, 27 patients (39%) were alive, while 42 patients (61%) had died. Among those still alive, 19 patients (28%) had durable remission with no further treatment required after GAMMA regimen all of whom had received it as second‐line therapy.

**FIGURE 2:**
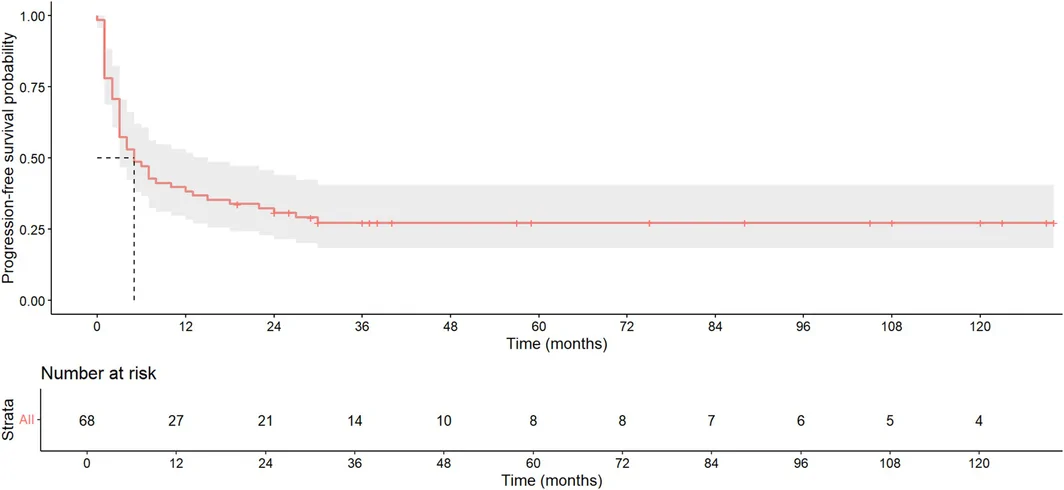
Kaplan–Meier curve of PFS for the overall cohort. The curve shows the progression‐free over time in months.

**FIGURE 3:**
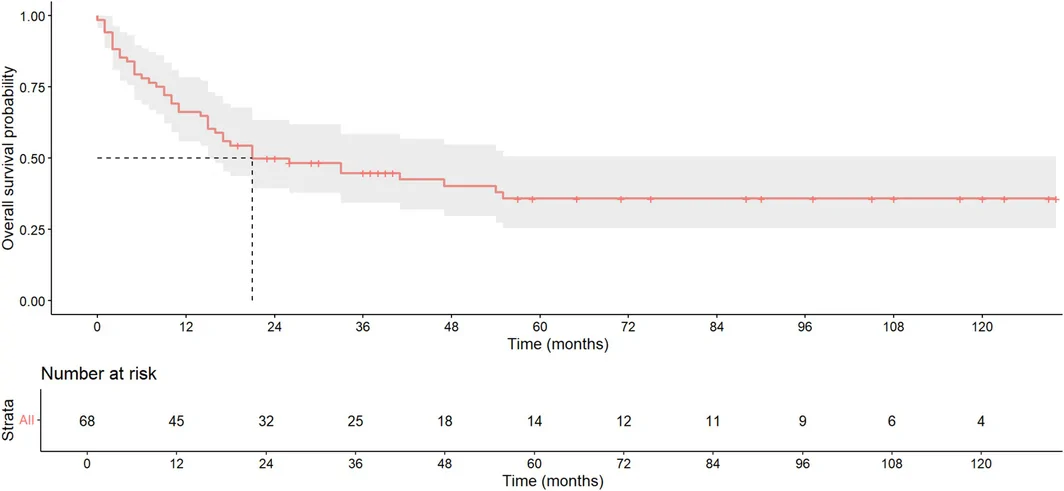
Kaplan–Meier curve of OS for the overall cohort. The curve shows the overall survival over time in months.

The 2‐year PFS rate was 31% (95% CI: 21%–44%) while the 2‐year OS rate was 49% (95% CI: 39%–63%). These outcomes were assessed in a total of 68 patients and are illustrated in Figures 2 and 3.

**FIGURE 4:**
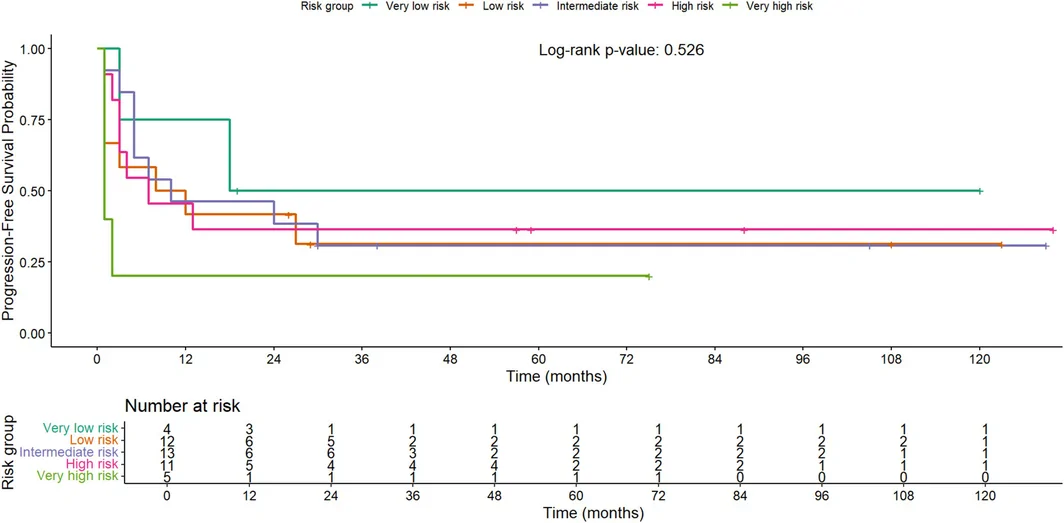
Kaplan–Meier curves for PFS stratified by IPFSG risk group. The plot illustrates PFS over time in months for patients in the very low, low, intermediate, high and very high IPFSG categories.

The PFS and OS at 2 years were also stratified according to IPFSG risk groups. These analyses were conducted in 61 patients as 7 patients did not have documented IPFSG scores. The 2‐year PFS rates were 50% (95% CI: 19%–100%) for the very low‐risk group, 42% (95% CI: 21%–81%) for low‐risk, 39% (95% CI: 19%–77%) for intermediate‐risk, 36% (95% CI: 17%–80%) for high‐risk and 20% (95% CI: 4%–100%) for very high‐risk patients (*p* = 0.526). These are illustrated in Figure 4 and Table 4.

**TABLE 4:** PFS at 2 years stratified by IPFSG risk group.

IPFSG group	2‐year PFS	95% CI, *p* = 0.585
Very low	50%	19%–100%
Low	42%	21%–81%
Intermediate	39%	19%–77%
High	36%	17%–80%
Very high	20%	4%–100%

**FIGURE 5:**
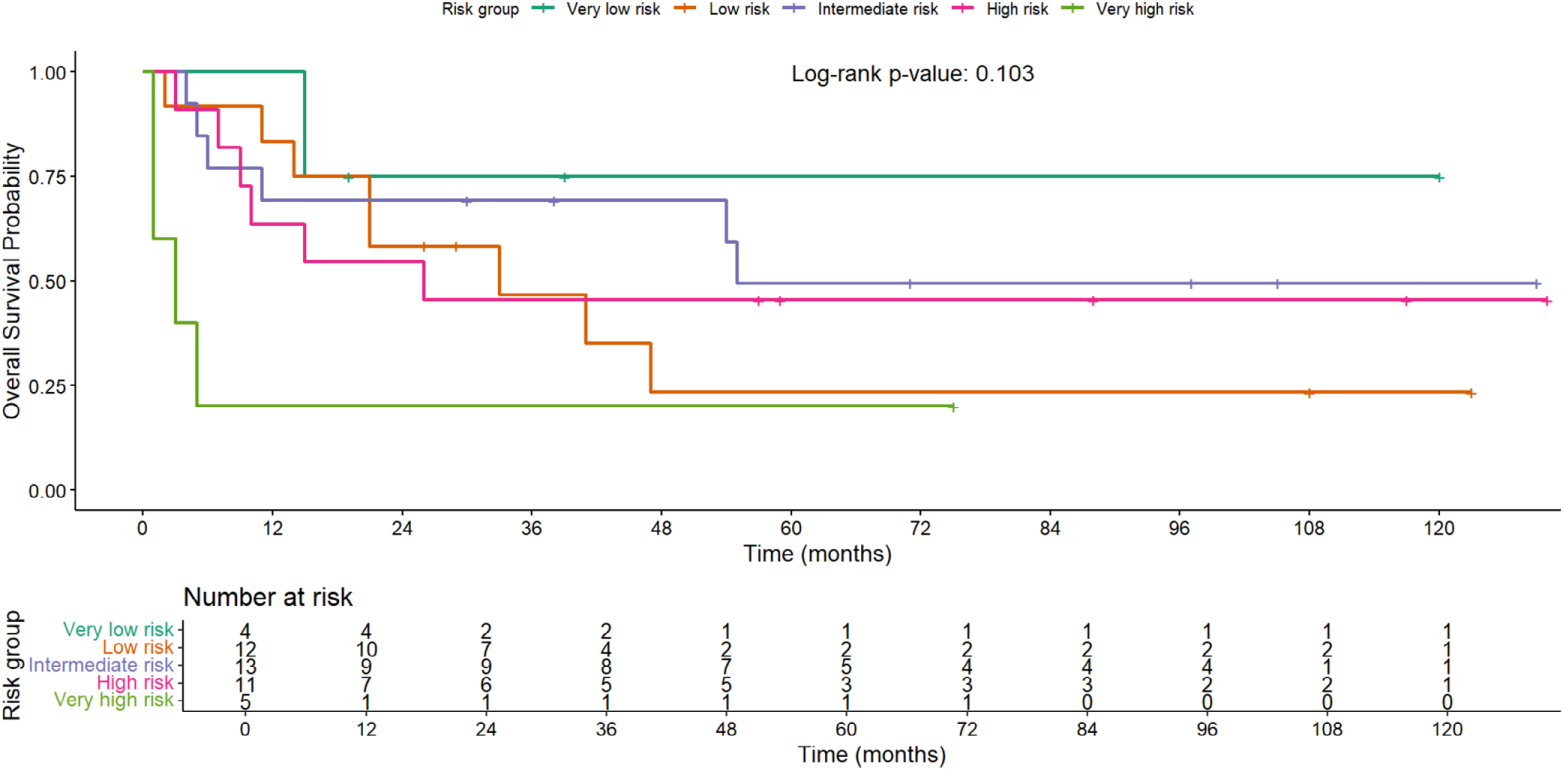
Kaplan–Meier curves for OS stratified by IPFSG risk group. The plot illustrates PFS over time in months for patients in the very low, low, intermediate, high and very high IPFSG categories.

The 2‐year OS rates were 75% (95% CI: 43%–100%) for the very low‐risk group, 58% (95% CI: 36%–94%) for low‐risk, 69% (95% CI: 48%–100%) for intermediate‐risk, 55% (95% CI: 32%–94%) for high‐risk and 20% (95% CI: 3%–100%) for the very high‐risk group (*p* = 0.103). These are illustrated in Figure 5 and Table 5.

**TABLE 5:** OS at 2 years stratified by IPFSG risk group.

IPFSG group	2‐year OS	95% CI, *p* = 0.169
Very low	75%	43%–100%
Low	58%	36%–94%
Intermediate	69%	48%–100%
High	55%	32%–94%
Very high	20%	3%–100%

**FIGURE 6:**
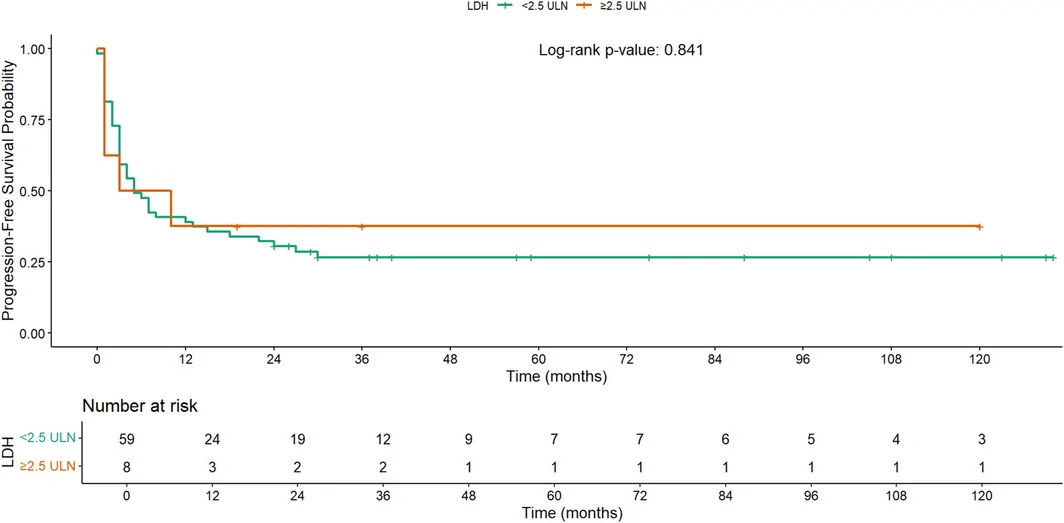
Kaplan–Meier curves for PFS stratified by baseline relapsed LDH levels. Patients were grouped based on LDH < 2.5xULN and ≥ 2.5xULN.

**FIGURE 7:**
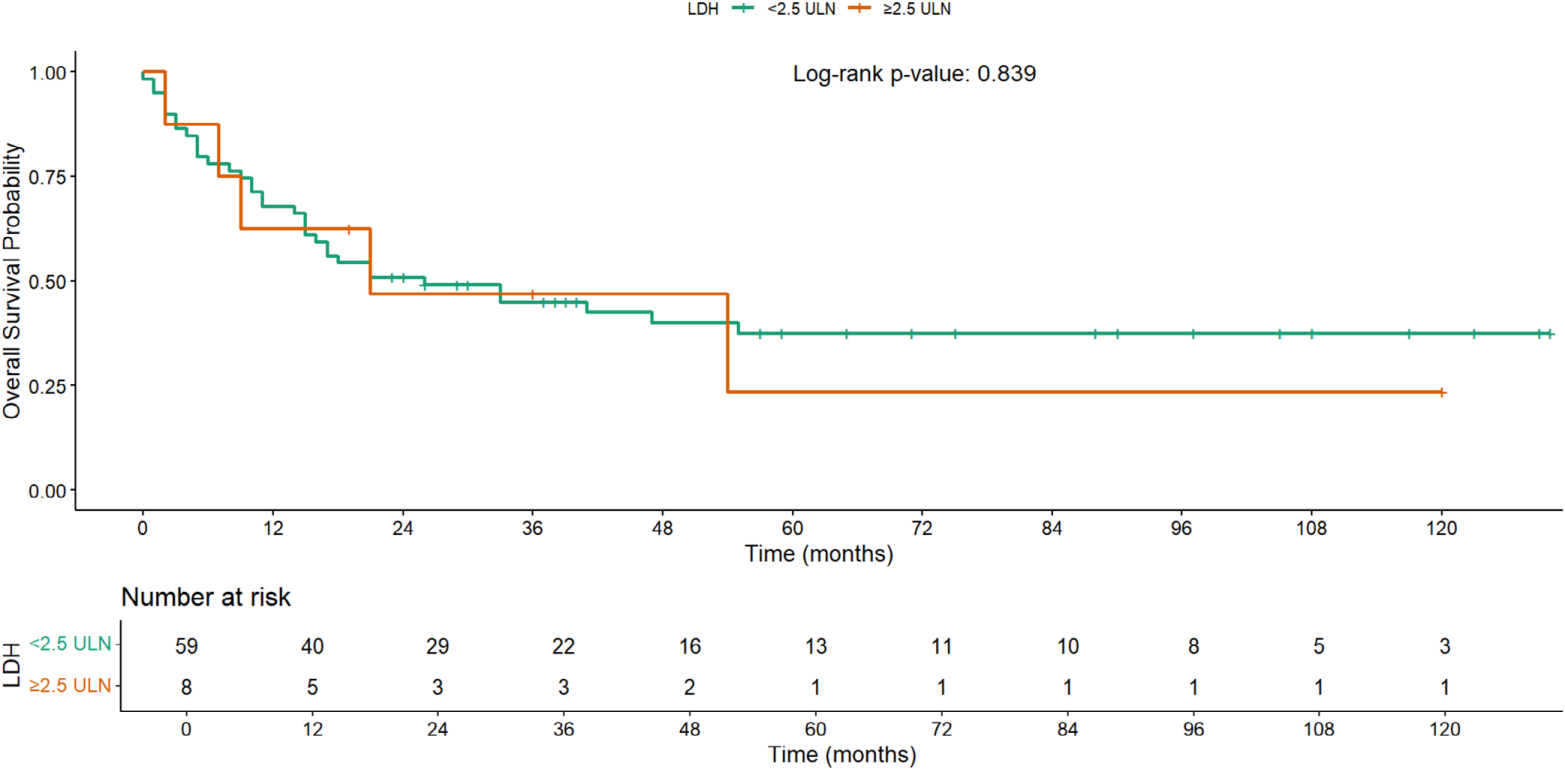
Kaplan–Meier curves for OS stratified by baseline relapsed LDH levels. Patients were grouped based on LDH < 2.5xULN and ≥ 2.5xULN.

The PFS and OS at 2 years were also analysed based on baseline relapsed LDH levels. Among patients with LDH < 2.5xULN, the 2‐year PFS rate was 31% (95% CI: 21%–45%), while the 2‐year PFS for patients with LDH ≥ 2.5xULN 38% (95% CI: 15%–92%) (*p* = 0.841). For OS, the 2‐year OS rate was 51% (95% CI: 40%–65%) in the LDH < 2.5xULN group and 47% (95% CI: 22%–100%) in the LDH ≥ 2.5xULN group (*p* = 0.839). These results are illustrated in Figures 6 and 7.

## 4 Discussion

There is currently no universally accepted standard of care regimen for salvage treatment in relapsed GCT. While some advances have been made, identifying effective and well tolerated salvage regimens continues to pose a clinical challenge. The GAMMA chemotherapy regimen has demonstrated promising activity in this setting, as reported in the phase II trial by Ng et al. [10]. This study extends those findings by including an additional group of patients treated outside of the clinical trial setting, resulting in the largest reported cohort to date receiving the GAMMA regimen.

In our study, the 2‐year PFS rate was 31% and the 2‐year OS rate was 49%. The ORR was 48%. Previous studies, such as McCaffrey et al. evaluated VIP or VeIP (vinblastine, ifosfamide and cisplatin) as first‐line salvage therapy and reported an ORR of 36% with a 2‐year OS of approximately 35%–40% [11]. Similarly, Loehrer et al. assessed the VeIP regimen in 135 patients and observed a 49.6% ORR with a 2‐year OS of 38% [12]. Both of these studies highlighted the particularly poor survival outcomes among patients with extragonadal non‐seminomatous germ cell tumours.

Alternative salvage strategies remain an area of clinical need, particularly for patients with cisplatin‐refractory disease or those with cumulative toxicity. While TIP (cisplatin, ifosfomide and paclitaxel) or HDCT remain widely used salvage regimens, their use may be limited in such patients due to nephrotoxicity and ototoxicity associated with cisplatin. The GAMMA regimen, incorporating oxaliplatin and paclitaxel, offers a non‐cross‐resistant alternative with a distinct toxicity profile, which may be advantageous in patients unsuitable for further cisplatin‐based therapy. Importantly, the high rate of successful stem cell mobilisation observed in this study (91%) supports its feasibility as a bridging regimen to HDCT.

Our study cohort was heterogeneous, including patients across the entire IPFSG risk spectrum. This contributed to the variation in outcomes with 2‐year OS ranging from 75% in the very low‐risk group to 20% in the very high‐risk group. Memorial Sloan Kettering Cancer Centre reported complete response rates of 70%–77% with the TIP regimen in relapsed GCT patients with favourable‐risk features [13]. However, a UK multicentre phase II trial by Mead et al. [14] also investigated TIP which enrolled a broader patient population and reported lower response rates (CR 19%, ORR 60%), highlighting the impact of patient selection and the importance of risk stratification when comparing outcomes across studies.

Elevated LDH was evaluated in our study as a prognostic marker and was associated with poorer outcomes. The 2‐year OS was 51% in patients with LDH < 2.5xULN, compared to 47% in those with LDH ≥ 2.5xULN with no statistically significant difference observed. These findings should be interpreted cautiously given the small number of patients with elevated LDH and wide confidence intervals. To our knowledge, this is the first analysis to examine the prognostic value of LDH specifically in the context of the GAMMA regimen. LDH has previously been assessed in the first‐line BEP setting, where elevated levels were associated with a decline in 3‐year OS from 97% in patients with LDH < 2.5xULN to 92% in those with LDH ≥ 2.5xULN [15]. Similarly, in a study of the carboplatin AUC10 regimen, 5‐year OS fell from 93% to 86% in patients with LDH ≥ 2.5xULN [16]. While LDH is an established adverse prognostic factor in other treatment settings, this study was underpowered to demonstrate a clear prognostic impact within the context of the GAMMA regimen.

The GAMMA regimen was generally well tolerated, with the majority of patients (64%) completing the planned course of treatment. This high rate of treatment completion suggests that the regimen is feasible in routine clinical practice. Treatment discontinuation due to toxicity occurred in 10% of patients. In the original GAMMA study in Ng et al., toxicity was reported to be manageable and predominantly haematological with the most common grade ≥ 3 adverse event was neutropenia which occurred in 26 patients (59%). Similarly, in a retrospective study from North‐West London examining patients who received curative‐intent chemotherapy for GCT, 69% experienced treatment‐related toxicity [17], yet the treatment discontinuation rate due to adverse effects was also relatively low at 15%.

Our study demonstrates that HDCT remains feasible after progression on the GAMMA regimen. In our cohort, 68% of patients proceeded to HDCT after successful stem cell harvest. Stem cell mobilisation was attempted in 34 patients, with success in 31 (91%) and failure in 3 (9%) and 20% remained alive and disease‐free. Mandachi et al. reported that stem cell mobilisation success rates are significantly higher when performed during first‐line therapy (93%) compared with second‐line therapy (71%) (*p* = 0.016) [18]. Our mobilisation success rate of 91% after GAMMA, predominantly given as second‐line therapy, supports its use as a first salvage option without compromising subsequent HDCT.

GAMMA provides an alternative treatment regimen particularly for patients with cisplatin‐resistant disease. The phase II trial by Kollmannsberger et al. demonstrated modest clinical activity of oxaliplatin even in heavily pretreated patients [19]. Oxaliplatin is associated with less nephrotoxicity and ototoxicity making it a more suitable option for patients with pre‐existing renal impairment or hearing loss. Its dose‐limiting toxicity is peripheral sensory neuropathy. Oxaliplatin has also been used in combination with agents like gemcitabine and paclitaxel showing synergistic antitumour activity [20, 21, 22]. The initial phase II study by Motzer et al. in 1994 demonstrated that paclitaxel has antitumor activity in patients with cisplatin‐refractory GCT [23]. Since then, paclitaxel has been incorporated into combination chemotherapy such as with gemcitabine, ifosfamide or cisplatin to enhance synergistic and improve efficacy [24, 25, 26]. These regimens have shown clinical activity in the salvage setting with some achieving durable response. Together, the oxaliplatin‐paclitaxel backbone of the GAMMA regimen offers a non‐cross‐resistant, tolerable alternative especially for patients who are cisplatin‐ineligible or have cumulative morbidity concerns.

This study has several limitations. As a single‐centre retrospective analysis, it lacks a comparator group, limiting the ability to directly compare the efficacy of GAMMA to other salvage regimens. The relatively small sample size and limited number of events precluded multivariable analysis, therefore limits the ability to adjust for potential confounders such as IPFSG risk group, prior lines of therapy and performance status. Our cohort was clinically heterogeneous with respect to prior treatment exposure, IPFSG risk category, performance status, and treatment line. While this reflects real‐world use of GAMMA, it limits interpretation and prevents robust conclusions regarding treatment effect. Analyses of post‐GAMMA treatments, including HDCT, are subject to potential immortal time bias, as only patients who survive and remain fit can proceed to subsequent therapy. Therefore, these findings should be interpreted as descriptive rather than comparative. Finally, the variability in follow‐up duration and censoring patterns may influence survival estimates, particularly at later time points, and should be considered when interpreting the reported 2‐year outcomes. Although Kaplan–Meier analysis accounts for censoring, these results should be interpreted with caution, especially in subgroup analyses with small sample sizes and wide confidence intervals.

## 5 Conclusions

To our knowledge, this study represents the largest reported series of patients with metastatic GCT treated with the GAMMA regimen. Our findings demonstrate that the GAMMA regimen is feasible and well tolerated in a heterogeneous and real‐world cohort. More than half of the patients were able to complete the full course of treatment with only a small proportion discontinuing due to toxicity. Stem cell harvest remained highly achievable after GAMMA, supporting the feasibility of subsequent HDCT. Importantly, this is the first study to evaluate the prognostic impact of LDH in the context of the GAMMA regimen, although no statistically significant associations was observed, the findings suggest a possible trend that warrants further investigations in larger studies.

## Author Contributions

**Kenrick Ng:** supervision, writing – review and editing, conceptualization, visualization. **Jonathan Shamash:** conceptualization, visualization, writing – review and editing, supervision. **Prabhakar Rajan:** conceptualization, writing – review and editing, visualization, supervision. **Nasreen Abdul Aziz:** conceptualization, writing – original draft, writing – review and editing, formal analysis, data curation, visualization.

## Funding

The authors have nothing to report.

## Ethics Statement

This retrospective study was registered and approved by the Clinical Audit Department of St Bartholomew's Hospital (project numbers 14,581).

## Conflicts of Interest

The authors declare no conflicts of interest.

## Data Availability

The data that support the findings of this study are available from the corresponding author upon reasonable request.
